# Epidemiological Assessment and Inference in Race-Based Clinical Algorithms: A Narrative Review and Health Policy Analysis Focused on Living Kidney Donation

**DOI:** 10.1089/heq.2024.0170

**Published:** 2025-03-11

**Authors:** Sienna E. Schaeffer, Carolina Gonzalez Bravo, Christopher D. Ahlers, Alaina N. Elliott-Wherry, Hannah Zadeh, Precious-Junia de-Winton Cummings, Kimberly C. Dukes, Nasrien E. Ibrahim, DeShauna Jones, Patrick T. Zamba, Aloha D. Wilks, Martha L. Carvour

**Affiliations:** ^1^ Department of Internal Medicine, University of Iowa Carver College of Medicine, Iowa City, Iowa, USA.; ^2^ Department of Community and Behavioral Health, University of Iowa College of Public Health, Iowa City, Iowa, USA.; ^3^ Department of Sociology and Criminology, University of Iowa College of Liberal Arts and Sciences, Iowa City, Iowa, USA.; ^4^ Department of Epidemiology, University of Iowa College of Public Health, Iowa City, Iowa, USA.; ^5^ Division of Cardiology, Brigham and Women’s Hospital, Boston, Massachusetts, USA.; ^6^ University of Iowa Institute for Clinical and Translational Science, Iowa City, Iowa, USA.

**Keywords:** causal inference, epidemiology, evidence-based clinical practice, health equity, kidney transplant

## Abstract

**Background:**

Minoritized racial and ethnic groups in the United States face long-standing disparities in a variety of health outcomes, owing to inequitable distribution of social and structural determinants of health along racial and ethnic lines. Although the existence of such disparities has long been a topic of scientific inquiry, there has been a dearth of investigations regarding their underlying mechanisms and potential remedies. This presents a challenge to those creating evidence-based and equity-focused health policy.

**Methods:**

We conducted an evidence-based, equity-focused narrative review about living kidney donor eligibility using salient literature about donor eligibility and racial and ethnic disparities in kidney transplantation and donation in the United States. We sought to examine the rigor and reproducibility of the evidence base regarding race- and ethnicity-based living kidney donation policies.

**Results:**

Our review identified several threats to scientific validity in the evidence base, including ambiguity in the operationalization of race and ethnicity variables, instances of type III error and racial essentialist biases, and causal inferences made using underpowered or scientifically unsubstantial subgroup analyses. We also identified structural barriers to the interpretation of this evidence to advance health equity, including barriers to the practices of clinical equipoise and shared medical decision-making.

**Conclusions:**

Threats to scientific validity and inferential errors in the evidence base about health inequities may forestall progress toward equity. We provide recommendations for addressing such barriers using standards applied in other clinical and research domains.

## Introduction

Health disparities based on race, ethnicity, socioeconomic status, disability, sexual orientation, and gender identity have been well documented in the clinical and scientific literature for decades. Landmark accounts such as the *Report of the Secretary’s Task Force on Black & Minority Health* of 1985 and the Institute of Medicine’s 2002 report *Unequal Treatment: Confronting Racial and Ethnic Disparities in Healthcare* have summarized and subsequently inspired a large body of work documenting health inequities.^1,2^ For decades thereafter, calls have emerged from within academia and from within communities affected by health inequities to shift the focus from identifying disparities to actively reversing them. Reducing health disparities has been a target of the Healthy People initiative since Healthy People 2000 (originally penned in 1990),^3,4^ whereas the United States (U.S.) Department of Health and Human Services (HHS) released its own *HHS Action Plan to Reduce Racial and Ethnic Disparities* in 2011.^5^

Despite prevailing evidence that health disparities result from social and structural factors, such as disparate access to health care services, the translation of this knowledge into more equitable clinical policies and guidelines has been slow. Barriers to such translation may be rooted in the evidence base itself, as well as in users’ interpretation of it. Scientific frameworks that recapitulate disparities and, all too often, essentialize disparities as individual-level and unmodifiable traits persist in the extant literature,^6^ leaving clinicians and policymakers with few rigorously designed, equity-focused studies and even fewer interventional studies related to reversing inequities based on structural theories of race and racism.^7,8^ Such gaps in the literature may further perpetuate uncertainty about how to identify and implement equitable policies and compound organizational inertia across the U.S. public health and health care systems. In other words, although the evidence is clear that action is needed, gaps in and uncertainties about the health equity-related evidence base may serve as structural obstacles to action.

There have been notable, recent, and high-profile exceptions, such as the removal of race adjustments from estimated glomerular filtration rate (eGFR) calculations.^9^ eGFR is an instructive example for clinicians and policymakers throughout the field, not only because of its historical presentation as a race-stratified measure but because that measure has influenced other cascading aspects of care, including an individual’s eligibility for kidney transplantation or donation. For example, in 2022, the Organ Procurement and Transplantation Network (OPTN) established a requirement for transplant centers to transition to race-neutral methods for calculating eGFR.^10^ By 2023, transplant centers were advised to adjust wait list times for patients who were negatively impacted by the prior, race-based eGFR calculations. Although research on the outcomes of this change is still ongoing, OPTN reports that, after 2 years, there have been some positive intended changes, including a 3.5% increase in the number of Black transplant candidates who are listed as eligible for a transplant prior to dialysis and a 1.8% increase in the number of kidney transplants with Black recipients.^11^ These efforts are an important step to address long-standing racial disparities in kidney transplant access and outcomes in the United States.^12–15^ These efforts also show that, while inequitable policies may compound disparities, equitable policies may also lead to successive, structural measures to advance equity.^16^

Recently, we completed a policy-focused review of evidence surrounding kidney donation. Although Black Americans have higher rates of end-stage renal disease (ESRD) compared to White individuals, they are less likely to receive a transplant and less likely to have a living donor, which is associated with superior outcomes compared to transplantation from a deceased donor.^17–19^ In Purnell et al.’s analysis of living donor kidney transplantation between 2010 and 2014, after 2 years on the waitlist, 11.4% of White transplant candidates had received a living donor transplant, compared to 2.9% of Black patients.^18^ The rate of living kidney donation has decreased over recent years, whereas racial disparities have increased.^18,20^ Subsequently, a growing number of transplant centers have expanded eligibility for living kidney donation to hypertensive donors,^20^ even though hypertension may increase the long-term risks for chronic kidney disease among living kidney donors. However, donation eligibility has often been racially stratified: White individuals with hypertension may donate, whereas Black donors with hypertension may still be considered ineligible at many transplant centers.^15,20^

The goal of our review was to assess the rigor and reproducibility of limited evidence on this topic as well as identify any structural evidentiary barriers to advancing equity for living kidney donation. The review is intended to align with the OPTN’s recent guidance regarding eligibility for transplant recipients, as summarized above, by examining and informing policies regarding eligibility for transplant donors (such as living relatives of a potential recipient)—policies often established and overseen locally by transplant centers and addressing the patient populations and communities they serve. Our review of the salient literature and our synthesis of findings demonstrate several challenges in the contemporary evidence base that are not unique to kidney donation but that are frequently encountered in the health disparities literature. Thus, we elaborate on our findings and discuss approaches to overcoming these obstacles that persist in the evidence base across clinical and scientific disciplines.

## Materials and Methods

Many health centers previously considered race and ethnicity when determining eligibility for living kidney donation,^15,20^ based in part on prior reports about racial and ethnic disparities in health outcomes after donation. Our team conducted a policy-focused narrative review and synthesis, starting with a representative transplant center policy about eligibility for living kidney donation, evidence directly cited as guiding donation policies that apply racial and/or ethnic stratification,^21–23^ and broader evidence about disparities in access to kidney transplantation.^14,15,17–19^ Since key evidence cited in support of racially and/or ethnically stratified policies was identified in scientifically secondary components of the literature (e.g., subgroup analyses and/or discussion sections) rather than in studies explicitly designed or powered to address this topic, we sought to identify the context of the policy within the cited literature rather than to conduct a systematic or scoping review whose protocol would likely overlook this cited context (e.g., commentary in a discussion section not recognized by systematic search procedures). The reviewed literature represents both current discussions about living kidney donation eligibility published between 2018 and 2021 and historical kidney transplant data dating back to the 1960s.

Our multidisciplinary team included faculty, staff, and student leaders, whose collective expertise encompassed medical practice, population health science, clinical and translational science, health equity, health policy, health education, health systems improvement, community engagement, medical anthropology, and sociology. The evidence about racial and ethnic disparities in donor outcomes was collaboratively assessed through a series of equity-focused journal club-style sessions and collaborative written syntheses. As part of our review process, we acknowledged that the terms “race” and “ethnicity” have distinct meanings, yet this distinction may be blurred when ethnic groups are racialized and when health inequities are distributed across racialized ethnic groups. For example, Latine persons are often racialized in the scientific literature (e.g., failure to disaggregate by race, inclusion of Latine persons as the only ethnic group in an otherwise race-focused classification system).^24^

## Results

We identified several potential threats to scientific validity within the evidence base regarding race- and ethnicity-based stratification of transplantation guidelines and in the inferences drawn from this evidence (Table 1). First, the definitions of race and ethnicity variables in studies used to support such guidelines were typically absent or ambiguous. Meanwhile, race and ethnicity variables were often analyzed as individual-level traits signifying an exposure or risk, yet this approach was not, in turn, mapped onto scientific mechanisms explaining the relationship between race and ethnicity and the exposure or risk. By contrast, other variables depicting exposures or risks in the same studies (e.g., the definition of hypertension) were defined using clear and reproducible methods and tied to well-defined and modifiable mechanisms of health and disease. We noted that some of the literature regarding disparate risks across racial and ethnic groups was, in fact, presented in narrative discussions of study findings rather than within the study methods and results. In these cases, the scientific limitations of the resulting race- and ethnicity-based inferences were scarcely discussed.

**Table 1.** Threats to Scientific Validity and Potential Solutions Identified During a Narrative Review of Literature Regarding Race- and Ethnicity-Based Living Kidney Donor Eligibility Policies

Threats to scientific validity	Potential solutions
**Threat:** Ambiguity in definition and operationalization of race and ethnicity variables **Risk for:** Selection biases, misclassification biases **Example:** Race or ethnicity variable used to classify or stratify participants in a study without clear definition of how the variable was measured, modeled, or applied	Clearly define race and ethnicity as socially constructed variables and avoid using proxy terms which can obscure meaning and contribute to essentialism (e.g., “ancestry” or “heritage”). Define the theoretical frameworks within which race and ethnicity variables are being used and whether these variables serve as a proxy for other mechanisms (see row below).^24^
**Threat:** Use of race and ethnicity as essential, individual-level risk factors for disease and/or the conflation of individual risk factors with population-level, social, and structural mechanisms **Risk for:** Type III errors, racial essentialist biases **Example:** Race or ethnicity positioned in a multivariable model as an individual-level risk factor or confounder rather than a proxy measure for social or structural mechanisms that may mediate or moderate inequity	Elaborate on mechanisms underlying the relationship between race and ethnicity and an observed health inequity. Proposed mechanisms should be scientifically plausible, particularly regarding modern scientific understandings of race and ethnicity as socially constructed variables. Understand the fundamental limitations of searching for genetic explanations for racial or ethnic health disparities^25^ and avoid essentializing race and related variables. It may be necessary to consider adapting methodologies to elucidate mechanisms.^26^
**Threat:** Conclusions about differential subgroup experiences (e.g., racial subgroups) without conclusive evidence of such differences, proposed mechanisms for such differences, or statistical comparisons of the risks and benefits of alternate treatment scenarios or counterfactuals **Risk for:** Inferential errors, failure to recognize clinical equipoise **Example:** Racial or ethnic subgroup disparities applied as rationale for subgroup exclusion from an otherwise standard medical intervention due to purported disparities in the risk of that intervention without presentation of the counterfactual risk of the exclusion or of alternative, nonexclusionary measures to offset any purported disparities in risk	Maintain clarity about the scope of the research question and the limits of generalization. Elaborate fully on the risks and benefits of alternate or counterfactual treatment or policy scenarios considered. Engage patients and communities in health policies and health systems research. Recognize the best available evidence at the population level and discuss uncertainties, risks, and benefits with patients during shared decision-making.

Our review confirmed that, while Black or African American patients have been shown to have higher rates of ESRD compared to White patients, Black Americans are less likely to have a living donor and receive a transplant;^17,19^ and Black Americans who are referred as potential donors are more likely to encounter structural and/or clinical barriers to donation, including medical exclusions.^15^ Meanwhile, racial disparities in living donor transplantation have increased in recent decades.^18^

We found a relatively small body of evidence on the outcomes of living kidney donation among individuals from minoritized racial and/or ethnic groups, and statistical evidence or clinical concerns about racial or ethnic health disparities among living kidney donors tended to correspond with recommendations for heightened caution against donation for members of minoritized groups. However, this caution was generally unaccompanied by scientific investigations about how to reduce these disparities; and these recommendations were made without strong statistical evidence that denying donation would substantively improve (and not worsen) the observed health disparities.

## Discussion

Our review identified several gaps, uncertainties, and threats to scientific validity in the evidence base regarding race- and/or ethnicity-based stratification of living kidney donation policies. We examine these in detail below. We note that many of our key findings (e.g., ambiguous definitions of race and ethnicity variables in clinical studies) represent common themes encountered by researchers and policymakers across disciplines when identifying, explaining, and addressing racial and ethnic health disparities. Such barriers may forestall the implementation of evidence-based, equitable policies. To address these barriers more proactively, we propose that current best practices elsewhere in the clinical and scientific literature can help to overcome some of these barriers, and we elaborate further on these applications here.

### Ensuring scientific rigor and reproducibility when race variables are used

Our findings reinforce the importance of rigorous, reproducible, and scientifically responsible applications of race and ethnicity variables in research studies and in the clinical policies based on such research. In this review, we found that race and ethnicity variables in the supporting evidence base—and the interpretations and applications of race and ethnicity variables when that evidence was translated into practice—were neither clearly defined nor connected to mechanisms of health and disease. Such challenges are not unique to this area of the literature: Cross-national analyses of the evolution of census terminology, for instance, have demonstrated that racial and ethnic classifications are heterogeneous, complex, and inconsistently defined; and classifications are strongly influenced by variations within and across cultures over time.^27^ Likewise, in a review of over a decade’s worth of literature from the top 10 academic journals in general medicine, oncology, and surgery, it was found that 81 different classifications of race and ethnicity were used; and “these were often imprecise and open to interpretation.”^28^ Moreover, uses of race and ethnicity variables often lacked scientific rigor and reproducibility; less than 5% of articles provided a definition of race or ethnicity and just over 10% described how race and ethnicity data were classified.^28^

The failure to connect race and ethnicity variables to mechanisms by which disparities occur, while also prevalent in the extant literature about health disparities, forestalls scientific and clinical advancement by de-emphasizing the identification and evaluation of interventions that can reduce disparities. Furthermore, this approach may reinforce or exacerbate inequities by erroneously essentializing race and ethnicity as fundamental causal factors in health and disease.^23,24^ These approaches tend to neglect the complexity of constructs underlying race and ethnicity variables (and other socially constructed demographic variables) which can signify the inequitable distributions of social, economic, and health-related resources (sometimes called “structural racism”) that ultimately impact health outcomes.^26,29^ The harmful effects of racial essentialism have been well documented: Essentialist beliefs lead to indifference toward disparities among racial groups, decreased support for measures reducing social inequality, and less interest in interacting with members of racial outgroups.^30^ In scientific research, racial essentialism can lead to errors in the measurement of disparities on a population level and inferential errors in the interpretation of research results.^26^

One such inferential error is known as type III error, which occurs when a true association is found (to use the traditional language of error: we correctly reject the null hypothesis), but the association we detected answers a different question than the one posed.^31^ Research regarding health inequities tends to be prone to one form of type III error (Table 1)—that is, the conflation of individual-level risk factors (why do some individuals get the disease?) with population-level underlying causes of disease (why does the prevalence of the disease differ between groups of individuals?). One recurring example is the search for genetic explanations for racial health disparities. In the simplest of models, genetic variation at a single locus can explain differences in disease risk between two individuals living in the same environment. It can help answer the question, “Why do some people get the disease while others do not?” However, if we identify a genotype that is associated with disease, even if it is heterogeneously distributed between two groups, it would be a type III error to simply extrapolate that disparities between groups are related to the genotype unless a difference in distribution of the genotype between the two groups has been confirmed in unbiased, representative samples and the prevalence of the allele and the absolute risk associated with the genotype are quantitatively sufficient to explain or substantively contribute to the group-level disparity.

When it comes to racial health disparities, this endeavor is particularly fraught. The first requirement—that there be a difference in the distribution of the genotype between the two populations—can easily lend itself to racial essentialism by ascribing to the idea that there are specific genotypes that correspond to socially constructed racial groups. Even the premise of this question assumes the potential existence of clinically significant differences in the genetic composition of racial groups. Furthermore, this extrapolation relies on an overly simplistic model of a gene-to-disease relationship. The chronic diseases that are America’s leading causes of death are complex and multifactorial. The effect of any given gene and its various alleles on an individual’s overall health is mediated or moderated by their environment, which may include disparate experiences with social and structural health resources. Thus, observable phenotypes are typically the result of an individual’s genotype and interactions between their own genes, as well as their genes’ interactions with the environment. This reality presents a fundamental limitation to the search for genetic explanations for racial health disparities.^25^

Furthermore, we note that extensive research examining the purported genetic bases of racial and ethnic health disparities has had another important impact on scientific quality and population health: Such research tends to prioritize descriptive studies above rigorous mechanistic investigations. With time, this trend can further forestall meaningful scientific progress—shifting the focus away from equity-focused interventional research and potentially enshrining descriptive studies as sufficient support for exclusionary health policies. Proactive scientific efforts to advance equity might be better focused on ensuring that patients and communities have access to existing, well-proven clinical interventions and to informed, shared decision-making about these interventions.

### Recognizing patient autonomy when clinical equipoise is present

The evidence base is replete with pathophysiological and clinical information about the risks, benefits, and best practices for living kidney donation in general, including those ensconced in clinical practice guidelines on the topic.^32–36^ However, the contemporary evidence base provides scant information about the outcomes of living kidney donation among patients from minoritized racial and ethnic groups. In part, this scarcity of evidence may represent an inevitable consequence of race- and ethnicity-based exclusions affecting donor eligibility. Such policies reduce the number of donors which, in turn, reduces a health system’s ability to measure outcomes after donation. Indeed, one of the studies we reviewed commented specifically on this, noting a historic lack of inclusion of Black and Latine donors in studies on the topic, leading to a limited evidence base.^22^ Another study also acknowledged that the incidence of some adverse outcomes (such as ESRD) among donors is typically low, thereby reducing the statistical power to detect the effects of such outcomes, especially across underrepresented groups.^21^

Because statistical evidence across racial and ethnic subgroups is lacking, some researchers and policymakers have concluded for decades that the risks or harms of donation may be higher among minoritized racial and ethnic groups and that the inability to prove or disprove this assumption may justify exclusion from donor eligibility as a precaution (e.g., applying different restrictions for Black patients with hypertension compared to White patients with hypertension in whom other clinical characteristics, such as the number of medications used to treat hypertension, are otherwise equal). However, these approaches suggest that the absence of evidence about specific subpopulations may obviate the best available evidence on the population level and that safe and effective interventions must be proven in all individual subgroups before they can be safely or effectively applied. This is in contradiction to the standards applied to other clinical interventions, where medications and devices are frequently approved for use under appropriate clinical guidance based on outcomes measured in a subset of the population. Assertions that racial and ethnic subgroups are fundamentally different returns to the premise of essentialism described above. In other words, if outcomes are assumed to differ across racial and ethnic groups yet no modifiable mechanisms for such a purported difference are identified other than racial or ethnic identity, this approach aligns more with essentialist principles than with extant evidence. It prioritizes scientifically fraught assumptions about racial and ethnic differences above the existing and extensive evidence base about kidney donation.

We argue that this approach diverges not only from sound scientific and clinical reasoning but also from well-established ethical principles that guide clinical research and practice. Among these are the standards of clinical equipoise and the role of patient autonomy in medical decision-making (Table 1). Clinical equipoise refers to the state of an evidence base or of clinical investigators at the start of the clinical trial, for instance, wherein there is a genuine lack of consensus among experts as to whether an experimental treatment will differ from a standard treatment.^37^ Equipoise can also occur when experts disagree regarding best clinical practices, such as discordance in recommendations for hypertension treatment targets between the American College of Cardiology/American Heart Association, European Society of Hypertension, and the American Academy of Family Physicians.^38–40^ Medical ethicists recommend that when equipoise exists in a clinical setting, providers have a duty to share the uncertainty with the patient and engage in an in-depth discussion to allow them to participate in an informed decision. This standard still allows providers to offer clinically informed opinions and to exercise sound clinical judgment, particularly for highly complex, emergent, or potentially life-altering decisions.^41,42^ However, ethical standards consistently recognize that the autonomy of patients must be central to the decision-making process and that informed, shared decision-making includes a faithful representation of what is—or what is not—known, proven, or well established in the field.

Race- and ethnicity-based restrictions on living kidney donation may represent a systematic departure from this standard. In the absence of concrete evidence to confirm whether or how kidney donation might lead to disparately adverse outcomes, an assumption of disproportionate harm can lead, in turn, to decisions made on behalf of patients and entire patient groups, without diligent discussion of the risks or benefits of this decision, opportunities to reduce such risks, or the extent or quality of evidence on which the decision was based. Even when an intent to protect patients from undue (or disparate) harm is stipulated by such a policy, this is typically manifested by making the decision for—not in discussion with—affected patients or groups. Furthermore, the decision does not consistently or fully counterweigh the risks and benefits of undue (or disparate) harm resulting from reduced access to donation and transplantation procedures that the policy itself would enact.

### Prioritizing engagement over exclusion to advance scientific and clinical quality

As we have summarized above, the scientifically rigorous use of race and ethnicity data and the clinically ethical application of equipoise and autonomy all argue against racial or ethnic exclusion criteria in donor eligibility policies. Although removal of race- and ethnicity-based restrictions may increase access to living kidney donation and transplantation, we note that this step alone will not address the prevailing gaps in the evidence base needed to ensure equitable outcomes or foster the trust needed between patients, communities, and health systems to ensure that care is equitably delivered from hospitals to clinics to community settings. To achieve this goal and ensure equitable outcomes (including outcomes such as patient survival and ESRD-free survival after transplantation or donation), health systems can follow other best practices for ensuring that patients and communities, including historically excluded groups, are engaged in, informed about, and consulted about policies and protocols that may impact them^43,44^ (Table 1). Importantly, such engagement can facilitate a more robust understanding of the modifiable causes of adverse health outcomes, such as social determinants of health and unmet health-related social needs,^45^ affecting patients and communities served by a health system.^46^ In the scenario we have examined here, for living kidney donation, specific attention should be given to factors that may improve access to evidence-based primary and secondary preventive care for transplant donors as well as transplant recipients after a donation or transplant occurs. In turn, engagement of patients and communities can help to build more robust and more equitable clinical care systems; and this engagement can inform more rigorous and reproducible science—by enhancing equitable engagement in research studies, improving the accuracy of data used in research studies and inferences made from these data, and informing interventions that equitably advance care.

## Conclusions

Evidence-based policy and equity-focused policy are compatible goals, yet the evidence base pertaining to health equity contains multiple, frequently encountered gaps and uncertainties in the quality of the evidence and the inferences made from it. Here, we encountered several such limitations, including the presence of historical biases, racialized heuristics, and inferential errors. Our review of this topic has some important limitations itself, particularly that it is focused on a relatively narrow topic in clinical medicine where the literature is limited and where the policy landscape is variable. However, our review has some notable strengths—including the highly interdisciplinary approach to the topic, the dissection of factors influencing scientific rigor and reproducibility, and the potential extensions of our work to other health topics where inequities occur. In summary, we argue that the evidence base about health disparities does not exist in isolation from the rest of the clinical and scientific evidence base and that otherwise well-accepted standards of scientific reasoning and clinical ethics can and should be applied to the gaps and uncertainties encountered in the health equity literature. This approach can ultimately support shared medical decision-making with patients and communities, improve the scientific rigor and reproducibility of health equity-focused research studies, and bolster the evidence base with proactive, interventional research to address modifiable causes of health inequity.

## Abbreviations Used

eGFR: Estimated Glomerular Filtration Rate
ESRD: End-Stage Renal Disease
HHS: Department of Health and Human Services
OPTN: Organ Procurement and Transplantation Network
U.S.: United States
